# When “He” Can Also Be “She”: An ERP Study of Reflexive Pronoun Resolution in Written Mandarin Chinese

**DOI:** 10.3389/fpsyg.2016.00151

**Published:** 2016-02-12

**Authors:** Jui-Ju Su, Nicola Molinaro, Margaret Gillon-Dowens, Pei-Shu Tsai, Denise H. Wu, Manuel Carreiras

**Affiliations:** ^1^Basque Center on Cognition, Brain, and LanguageSan Sebastián, Spain; ^2^Ikerbasque, Basque Foundation for ScienceBilbao, Spain; ^3^Neuroscience of Language Laboratory, The University of Nottingham Ningbo ChinaNingbo, China; ^4^Graduate Institute of Translation and Interpretation, National Changhua University of EducationChanghua, Taiwan; ^5^Institute of Cognitive Neuroscience, National Central UniversityTaoyuan, Taiwan; ^6^Joint Research Center for Language and Human Complexity, The Chinese University of Hong KongHong Kong, Hong Kong; ^7^Department of Philology, University of Basque CountrySan Sebastián, Spain

**Keywords:** ERPs, reflexive pronoun resolution, type of gender information, gender specificity, Mandarin Chinese

## Abstract

The gender information in written Chinese third person pronouns is not symmetrically encoded: the character for “he” (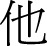, with semantic radical 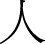, meaning human) is used as a default referring to every individual, while the character for “she” (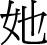, with semantic radical 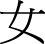, meaning woman) indicates females only. This critical feature could result in different patterns of processing of gender information in text, but this is an issue that has seldom been addressed in psycholinguistics. In Chinese, the written forms of the reflexive pronouns are composed of a pronoun plus the reflexive “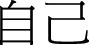/*self*” (

/*himself* and 

/*herself*). The present study focuses on how such gender specificity interacts with the gender type of an antecedent, whether definitional (proper name) or stereotypical (stereotypical role noun) during reflexive pronoun resolution. In this event-related potential (ERP) study, gender congruity between a reflexive pronoun and its antecedent was studied by manipulating the gender type of antecedents and the gender specificity of reflexive pronouns (default: 

/*himself* vs. specific: 

/*herself*). Results included a P200 “attention related” congruity effect for 

/*himself* and a P600 “integration difficulty” congruity effect for 

/*herself*. Reflexive pronoun specificity independently affected the P200 and N400 components. These results highlight the role of 

/*himself* as a default applicable to both genders and indicate that only the processing of 

/*herself* supports a two-stage model for anaphor resolution. While both reflexive pronouns are evaluated at the bonding stage, the processing of the gender-specific reflexive pronoun is completed in the resolution stage.

## Introduction

Anaphor resolution involves linking a given anaphor to a previously-mentioned antecedent in a sentence context, while interpreting both as related to the same discourse-level entity. Most studies related to the effects of gender information on anaphor resolution have proposed that (morpho)-syntactic rules constrain anaphor resolution (Carreiras et al., 1993, 1996; Garnham et al., 1995; Osterhout et al., 1997; Kennison and Trofe, 2003; Sturt, 2003; Duffy and Keir, 2004; Kreiner et al., 2009; Esaulova et al., 2014). From a theoretical perspective, anaphoric processing is considered to involve two stages of processing (Garrod and Sanford, 1994; Garrod and Terras, 2000). A bonding stage occurs first, for the purpose of searching for a best-fit referent among possible candidates (i.e., the antecedents) based on information related to gender, number, and syntactic rules. A resolution stage subsequently accomplishes the interpretation of the anaphor, taking into account world knowledge, and contextual information.

There is debate, however, about the degree to which lexical-semantic and syntactic cues are employed to resolve the anaphor. Carreiras et al. (1996) studied what linguistic information constrains pronoun resolution processing, by employing stereotypical role nouns as antecedents. They measured English and Spanish speakers' self-paced reading times and found cross-linguistic differences for the different morphological gender marking systems in English and Spanish. According to these authors, as soon as the mismatch of stereotypical gender is detected by the participants (i.e., on the pronouns encountered after stereotypical role nouns in English or on stereotypical role nouns at the beginning of a sentence in Spanish), it immediately influences processing, resulting in different patterns during pronoun resolution. Eye-tracking studies using stereotypical role nouns as antecedents (Sturt, 2003, in English; Esaulova et al., 2014, in German) have supported a two-stage model of anaphor resolution and these authors interpreted their findings as evidence for the syntactic constraints employed in resolving the link between an anaphor and its antecedent at the initial stage. Esaulova et al. (2014) concluded: “*anaphor resolution […] seems to depend above all on the rules of grammatical agreement in the context of overlapping gender cues*” (p. 798).

Osterhout et al. (1997) carried out an ERP study in English, in which the gender type of the antecedent (definitional vs. stereotypical) and the antecedent-reflexive pronoun gender congruity were manipulated (see also Kreiner et al., 2009 for an ERP study and Kreiner et al., 2008 for an eye-tracking study). While definitional role nouns had a definite gender (e.g., mother/father), gender in stereotypical role nouns was inferred based on world knowledge (i.e., a role noun that could refer to two genders but is biased toward one, e.g., electrician is male-biased and beautician is female-biased). The results showed similar P600 effects for antecedent-reflexive pronoun gender mismatches in conditions of both definitional (*mother—himself*) and stereotypical (*nurse—himself*) role nouns. These authors concluded that gender information was grammatically encoded even for stereotypical role nouns. Based on their interpretation, it would follow that anaphor resolution correlates with a single ERP component, the P600, and engages a process that is syntactic in nature rather than semantic/pragmatic. However, Nieuwland and Van Berkum (2006) found an N400 effect for pronouns with antecedents of the same gender as compared to those with antecedents of different gender in a Dutch study and attributed the results to differences in the contextual bias that would modulate the N400 effect (used to index semantic/context related processing) during anaphor resolution. Results obtained from German studies are heterogeneous. Schmitt et al. (2002) investigated ERP responses to pronouns related to biological (definitional) and grammatical gender entities. The authors claimed that anaphor resolution is basically syntactically driven (P600 effect found) but can interact with semantic information in the N400 time interval. Irmen et al. (2010) focused on the link between antecedent stereotypical gender and anaphor lexical-semantic gender (these men/women/people) in German. They reported an N400 stereotypical gender effect and a P600 effect for anaphor mismatch with the antecedent's stereotypical gender. These authors interpreted their findings as supporting the two-stage model: while stereotypical gender information is collected in the bonding stage, the resolution stage represents integration, driven by either lexical-semantic mismatch, or syntactic violation on the anaphors.

Results from Chinese ERP studies also reveal somewhat different patterns. Qiu et al. (2012) manipulated the distance and gender congruity between antecedent and pronoun in Chinese sentences. N400 and P600 mismatch effects were found respectively for short and long distance manipulations. These authors claimed that the processing of gender information in Chinese pronoun resolution is more “semantics-based” when the pronoun is closer to the antecedent and this representation decays as the distance increases. Xu et al. (2013) (Experiment 1, singular antecedent) also manipulated gender congruity between antecedent and pronoun across clause boundaries (i.e., long-distance dependency). Only P600 effects were reported for gender mismatches. However, the authors did not interpret this P600 effect as reflecting purely syntactic processing, but either semantics-based processing -computation of the semantic relationship between antecedent and anaphor- or general integration difficulty resulting from conflicts on gender during Chinese anaphor resolution. It is important to note, however, that in these two Chinese studies, the gender specificity of the pronouns (i.e., 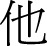/*he* and 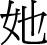/*she*) was considered to be symmetrical, as is the case in morphological gender languages. However, co-reference processing between the anaphor and the antecedent could in fact differ in Chinese, due to the asymmetry of gender specificity encoded in the Chinese characters for these pronouns.

As can be seen from the research reported above, the processing of gender information has been investigated by employing the co-indexation structure of anaphor resolution mainly in languages in which the morpho-syntactic gender marking on anaphor^1^ is “symmetrically” expressed, i.e., “*he/himself*” is used specifically for male antecedents and “*she/herself*” exclusively refers to female antecedents. However, in a language without inflectional morphological gender markings, such as Chinese^2^, the gender specificity is not symmetrically encoded in the written forms of the pronouns. In the spoken language, the third person singular pronoun is pronounced the same, /tā/, for both genders and the gender of a pronoun is inferred based on the context. Gender distinction is thus made only in written Chinese. Although the characters for the male and female pronouns share the same phonological component “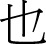,” they differ in their semantic radicals^3^. According to the web-based Dictionary of Chinese Character Variants^4^ established by the Ministry of Education in Taiwan, the character 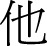/*he*, which contains the semantic radical “
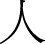
” (/rén/*human*) is the “third person pronoun, refers to a third person” and the character 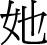/*she*, pronounced as /tā/, containing the semantic radical “
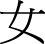
” (/nǚ/*woman*) is the “female third person pronoun^5^.” When reading Chinese, therefore, during anaphor resolution it could be the case that the relevance of the gender information provided by an antecedent may differ depending on the extent to which gender information is specifically presented or not in the anaphoric pronoun. The processing of the anaphor may also differ when the antecedent's gender is either definitional, with a clear gender, or stereotypical, where gender can only be inferred. This critical feature (i.e., asymmetry of gender specificity in Chinese pronouns) has rarely been tested in previous Chinese studies and could result in distinct patterns of processing of gender information.

We illustrate below why the gender specificity encoded in the pronouns is not likely to be symmetrical, from three different perspectives: the historical background of the characters, the difference in their semantic radicals, and the usage of 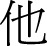/*he* as a default. First, from a historical point of view, 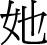/*she* was only recently proposed as the third person singular female pronoun by a linguist, Liu in 1921 for the convenience of translation from western languages (Ling, 1989; Chang, 2007; Hua, 2012)^6^. Previous to this, the default 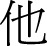/*he* was used in written Chinese. The use of the character 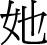/*she* was not generally accepted at first and even now its appearance and necessity remains controversial (Moser, 1997; Chang, 2007; Wang, 2010; Hua, 2012). In recent years, due to campaigns for gender equality, the two pronouns have been gradually differentiated but the use of 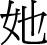/*she* is still not compulsory for female antecedents (Peng, 2009).

Secondly, the semantic radicals encoded in the two pronouns play a critical role in bringing out the gender specificity. In Chinese, the radical 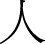/rén/ means *human* and the radical 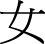/nǚ/ means *woman* and these different semantic radicals make the characters orthographically distinct from each other. Increasing evidence has shown that Chinese speakers rely very much on sub-lexical units -semantic radicals and phonological components- during text comprehension (Perfetti and Zhang, 1991; Feldman and Siok, 1999; Ho et al., 2003; Liu et al., 2003; Ding et al., 2004; Lee et al., 2007; Hsu et al., 2009; Tsang and Chen, 2009). A study carried out by Cherng et al. (2009), which explored whether Chinese script reflects negative attitudes toward women (whether characters containing the semantic radical for “woman” have a more negative valence), found no evidence of this in Chinese speakers' perception of gender-based characters. They reported no negative attitudes toward characters containing the semantic radical for “woman” (the meaning conveyed by the characters was rated by participants as positive, negative or neutral). However, while characters containing the radical 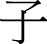/zǐ/*son* and radical 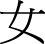/nǚ/*woman* were rated as positive, characters with the radical 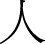/rén/*human* were rated as neutral. These results may imply differences in the mental representations of the gendered semantic radicals, especially when they appear in pronouns denoting different gender specificity.

Third, from an empirical point of view, the asymmetry on gender specificity presented by the pronouns is reflected in the usage of 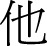/*he* as a default. Wu and Liang (2008) analyzed 150 news items taken from the Academia Sinica Balanced Corpus (ASBC), for a rule-based corpus analysis of Chinese pronominal anaphor resolution. The results showed an error rate of 0.21 for gender mismatches between 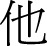/*he* and female antecedents. The authors attributed this relatively high rate of mismatch to the use of 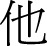/*he* as default in Chinese written text. Different learning sequences of the two pronouns at school also contribute to the tendency to use 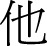/*he* as a default. A textbook analysis carried out by Huang and Luh (2012) in Taiwan reported that while children learn 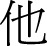/*he* in the first year of elementary school, 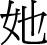/*she* is learnt in the second year. In some textbook articles, 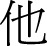/*he* is used to refer to female antecedents before and even after the pronoun 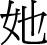/*she* is learnt. Word frequencies of the pronouns reported by Academia Sinica (Word List with Accumulated Word Frequency in Sinica Corpus^7^) correspond to such usage trends in reality (see Table 1).

**Table 1:** **Word frequencies for the two Chinese pronouns extracted from Word List with cumulated Word Frequency in Sinica Corpus, Academia Sinica, Taiwan**.

	** 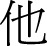 /*he***	** 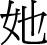 /*she***
Cumulative Word Frequency for Modern Chinese words (based on the corpus size of 5 million words)	29,938	10,755
Cumulative Word Frequency for Pre-modern Chinese Corpus	37,259	2
Cumulative Word Frequency for Old Chinese Corpus	36	Word not found

Data from these three perspectives thus clearly indicate that gender specificity in the characters for Chinese third person pronouns is non-symmetrical. Investigating this asymmetry can shed light not only on the processing of gender information in written Chinese but also on the general processing of pronouns in text.

In the present study, we investigate how the asymmetry of gender specificity interacts with antecedent noun type in which the biological gender is differently inferred during anaphor resolution in written Chinese. The experiment had a 2 × 2 × 2 design, with three factors manipulated: antecedent gender type (definitional vs. stereotypical), reflexive pronoun gender specificity (default vs. specific), and gender congruity between the reflexive pronoun and antecedent (congruent vs. incongruent).

First, antecedent gender type was manipulated. For definitional gender, because most female definitional role nouns in Chinese carry a 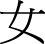/nǚ/*woman* radical in the same position as 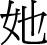/*she*, (such as 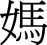/mā/*mother*, 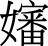/shěn/*aunt*, and 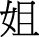/jiě/*elder sister*, Tang, 1988), proper names were used, to limit priming effects due to the presence of the same semantic radical in antecedent and reflexive pronouns (see Feldman and Siok, 1999; Ding et al., 2004). So, proper names such as 
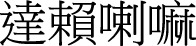
/*Dalai Lama* or 
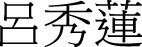
/*Annette Lu* were used as antecedents for definitional gender. Stereotypical role nouns (e.g., 
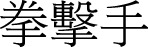
/quán jī shǒu/*boxer*, male-biased; or 
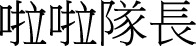
/lâ lâ duì zhǎng/*cheerleader*, female-biased) were used as antecedents for the stereotypical gender condition.

Second, gender specificity (default vs. specific) was manipulated on reflexive pronouns instead of personal pronouns. In Chinese, the use of pronouns is not constrained by the local structure (Principle B, Chomsky, 1981: pronouns cannot co-refer with antecedents in a local clause). A reflexive term, 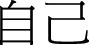/zì jǐ/*self* is allowed to appear after a pronoun to make it a reflexive pronoun co-referential to the subject in the same clause (Principle A, Chomsky, 1981), (see Li and Thompson, 1981). For instance, in (1a) the 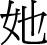/*she* could refer to the teacher or another female. In (1b), the reflexive, 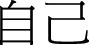/zì jǐ/*self*, helps to make the pronoun unambiguously co-referential to the previously mentioned animate antecedent (i.e., Mary) in the same clause (i.e., local binding of 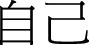/zì jǐ/*self*, see Jäger et al., 2015). Therefore, to avoid any confusion in co-reference between an anaphor and its antecedent, the reflexive 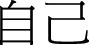/zì jǐ/*self* was added after the third person pronouns to form the third person reflexive pronouns (

/tā zì jǐ/ *himself*, default; and 

/tā zì jǐ/ *herself*, specific).

(1)
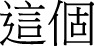

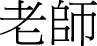

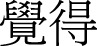
 Mary 
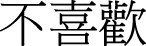

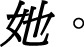
    /zhè gè/ /lǎo shī/ /jiào dé/ /Mary/ /bú xi huān/ /tā/
    (This teacher thinks that Mary doesn't like ***her***.)
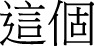

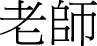

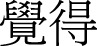
 Mary 
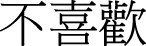

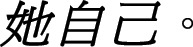
    /zhè gè/ /lǎo shī/ /jiào dé/ /Mary/ /bú xi huān/ /tā zì jǐ/
    (This teacher thinks that Mary doesn't like ***herself***.)

Third, we manipulated gender congruity between a reflexive pronoun and its antecedent (congruent vs. incongruent). It should be noted that when 

/*himself* appears after a female/female-biased antecedent, the sentence might still be acceptable in Chinese because 

/*himself* can be used as a general term referring to both genders, even though we consider this as a mismatch in the data analysis. An ERP mismatch paradigm was employed.

ERPs (Event-Related Potentials) are scalp recordings of electrical brain activity time-locked to a stimulus event. Compared to other neuroimaging techniques, they offer very good temporal detail of brain activity. This makes the ERP technique one of the best measures for disentangling the temporally incremental neural processes typically assumed by cognitive models. In ERP studies related to anaphor resolution, three major correlates have been discussed (see Callahan, 2008): the LAN (Left Anterior Negativity), the N400, and the P600. The LAN is a negative-going wave mostly observed at left anterior scalp electrodes from around 250–500 ms post target word onset. The LAN is related to automatic parsing involving morphosyntax and rule-based decomposition processes (Hahne and Friederici, 2002; Barber and Carreiras, 2005; Molinaro et al., 2008a,b, 2011; Hagoort, 2009). The N400 is a negative-going wave peaking around 400 ms after the onset of the target word, mainly recorded from electrodes in the centroparietal scalp regions (Kutas and Hillyard, 1980; Molinaro et al., 2010). The N400 is thought to represent context-dependent, lexico-semantic processing of a given stimulus. Its amplitude can be modulated depending on the lexical properties of single words and, at the sentence level, the anticipation/contextual semantic fit of a word with the previous context (Kutas and Federmeier, 2011, for a review). The P600 effect is a positive-going wave observed ~500–700 ms after target word onset, with centroparietal scalp distribution. This component was initially reported as correlating with (morpho-) syntactic violations (Osterhout and Holcomb, 1992). Recently, however, the interpretation of the P600 effect has been extended to reflect more general (also semantic) integration difficulties during sentence processing (Münte et al., 1998; Kaan et al., 2000; Kaan and Swaab, 2003; Carreiras et al., 2004; van Herten et al., 2005; Van de Meerendonk et al., 2009; Brouwer et al., 2012; Molinaro et al., 2012). In addition to these three components, the P200 component reflects a wide range of attention-related feature analysis including color, orientation and size of a feature (Luck and Hillyard, 1994). It is reported in studies related to word frequency (Dambacher et al., 2006), syllable frequency/structure (Barber et al., 2004; Carreiras et al., 2005), and Chinese character recognition (graphic, semantic, or phonological) (Liu et al., 2003; Lee et al., 2007; Hsu et al., 2009; Yum et al., 2014). Critically, the P200 component has recently been reported in Chinese discourse inference (Hung and Schumacher, 2012, 2014) and is considered to be related to a certain expectation driven by the context. It is reported as distributed mostly in the anterior region; the more positive the P200 amplitude, the less familiar, lower frequency and less expected the target word.

Based on previous ERP studies and the two-stage model of anaphor resolution (Garrod and Sanford, 1994), interactions among antecedent gender explicitness, reflexive pronoun gender specificity, and gender congruity are thus expected mainly in the N400 or P600 time intervals. Since the pronoun 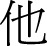
*/he* could be considered as a default pronoun (that can refer to both male and female antecedents) (Wu and Liang, 2008) and supported by the word frequencies of both pronouns in the Sinica Corpus, two hypotheses are possible about how the default pronoun is perceived for gender. First, if the 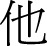
*/he* is recognized as male-biased, a gender mismatch N400 or P600 effect is predicted for 

*/himself*. Second, if the 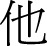
*/he* is understood as equally applicable to both genders, no gender mismatch effects are expected for 

*/himself* following female or female-biased antecedents (e.g., 
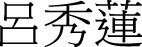
/*Annette Lu* or 
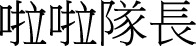
/*cheerleader*). Whether or not the second hypothesis results in a mismatch effect is considered a key result in the present study, to evaluate participants' sensitivity to the gender asymmetry of the reflexive pronouns. On the other hand, stronger gender mismatch effects are expected for the female specific reflexive pronoun, 

/*herself*, because this contrast (male/male-biased antecedent vs. specific female reflexive) could lead to strong gender incongruity. This contrast is also critical in evaluating how the specific reflexive pronoun is processed depending on the gender type of the antecedent. Both N400 and P600 effects are expected in this “pure” mismatch gender contrast, reflecting semantic/pragmatic processing costs (Osterhout et al., 1997; Callahan, 2008; Tsai et al., 2009; Irmen et al., 2010; Molinaro et al., 2012; Qiu et al., 2012; Xu et al., 2013). For the relatively less familiar and lower frequency orthographic form (i.e., the specific reflexive pronoun 

*/herself*), the attention-related P200 effect (Luck and Hillyard, 1994; Liu et al., 2003; Hung and Schumacher, 2012, 2014; Lee et al., 2012) and frequency-related N400 effect (Kutas and Federmeier, 2011) are also expected. Specifically, we are interested in the time course of the ERP effects, to see when and how the linguistic sources of gender information denoted by the reflexive pronouns interact with antecedent gender type and gender congruity during reflexive pronoun resolution.

## Materials and methods

### Participants

Forty native Chinese speakers (20 males, mean age: 21.8, aged 20–36 years) were recruited from the National Central University, Taiwan and were paid for their participation. They were healthy, right-handed, with normal or corrected-to-normal vision and reported no neurological or psychiatric history. The design and execution of the experiment conformed to the ethical regulations of the Institute of Cognitive Neuroscience at National Central University in Taiwan, which are equivalent to international standards. Informed consent was obtained from each participant.

### Materials

Proper names of eighty celebrities (40 males) were selected from the news, based on Google search from July to September 2011. The names chosen had a number of occurrences larger than 300,000. Eighty sentences related to the 40 male and 40 female celebrities were used. Half of the 40 sentences containing the male/female proper names had a congruent third person reflexive pronoun and the other half had an incongruent one. These sentences were the experimental materials for the condition of antecedents with definitional gender.

The stereotypical role nouns were selected based on the results of a questionnaire containing 348 generic role nouns (e.g., 
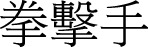
/quán jī shǒu/*boxer*, 
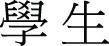
/xué shēng/*studen*t, or 
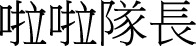
/lā lā duì zhǎng/*cheerleader*). Participants were asked to mark on an 11-point Likert scale from 0 to 100% with the percentage of 10 as the scale interval. The point of 0%-male/100%-female was always on the left and 100%-male/0%-female was always on the right. Fifty-seven college students (14 males, mean age: 21.58) from Tsing Hua University and Sun Yat-Sen University filled in this questionnaire. The forty most male-biased role nouns and 40 most female-biased ones were used as antecedents with stereotypical gender in 80 sentences. Half of the male-biased/female-biased antecedents were associated with a congruent third person reflexive pronoun and the other half with an incongruent one in sentences. The sentence structure for all materials was constructed as short-distance [i.e., the antecedent and the reflexive pronoun were in the same clause. See (2) and (3)]. At the beginning of each sentence, a segment denoting a time, location, or circumstance appeared. This was followed by a clause with S+V+O structure. The subject (i.e., antecedent) was presented by means of a proper name or stereotypical role noun. The object (i.e., anaphor) was the reflexive pronoun referring back to the subject. The target word was always the reflexive pronoun in each sentence, located in the fourth, fifth, or sixth position and never appeared AT THE END of the sentence.

(2) 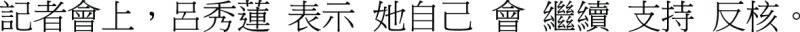/jì zhě huì shàng/, /Annette Lu/ /biǎo shì/ /tā zì jǐ/ /huì/ /jì xù/ /zhī chí/ /fǎn hé/(In the press conference, Annette Lu expressed ***herself*** about continuing to support the anti-nuclear movement.)
(3) 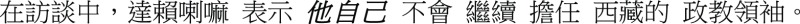/zài fǎng tán zhōng/, /Dalai Lama/ /biǎo shì/ /tā zí jǐ/ /bú huì/ /ji xù/ /dān rèn/ /xī cáng de/ /zhèng jiāo ling xiù/(In the interview, the Dalai Lama expressed ***himself*** about not continuing to serve as the political religious leader in Tibet.)

An additional 80 filler sentences were created. The manipulations on critical words were focused on whether their semantic meaning could fit into the sentence or not (40 semantic match vs. 40 semantic mismatch). Data from these 80 sentences were not included in the data analysis. In total, 240 sentences were employed in this study. Thirty percent of the sentences were accompanied by comprehension yes/no questions (i.e., 72 questions) to evaluate participants' understanding of the sentences. The questions were related to the description of the main character and never related to any gender information of our interest. List 1 contained all the 240 sentences mentioned above. For counter-balancing purposes, a second list was created. List 2 contained the same 240 sentences as in List 1 but with all the target words presenting the opposite manipulation (see Table 2 for examples of materials) (those items used were listed as Supplementary Material available online).

**Table 2:** **Example sentences used in the experiment**.

	**Default reflexive pronoun, 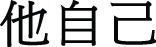 /*himself***	**Specific reflexive pronoun, 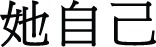 /*herself***
**ANTECEDENT WITH DEFINITIONAL GENDER**		
**Gender congruent**	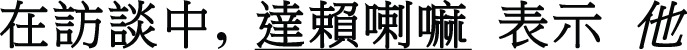 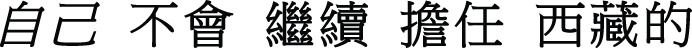 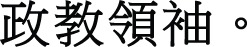 (20 sentences)(*In the interview, the **Dalai Lama** expressed **himself** about not continuing to serve as the political religious leader in Tibet.*)	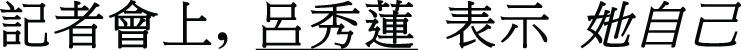 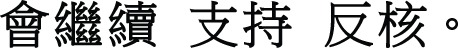 (20 sentences) (*In the press conference, **Annette Lu** expressed **herself** about continuing to support the anti-nuclear movement.*)
**Gender incongruent**	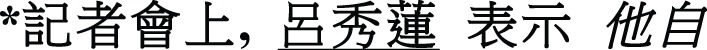 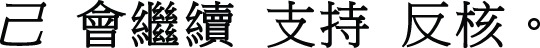 (20 sentences)	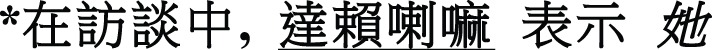 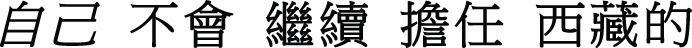 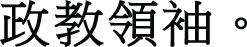 (20 sentences)
	(^*^In the press conference, **Annette Lu** expressed **himself** about continuing to support the anti-nuclear movement.)	(^*^In the interview, the **Dalai Lama** expressed **herself** about not continuing to serve as the political religious leader in Tibet.)
**ANTECEDENT WITH STEREOTYPICAL GENDER**		
**Gender congruent**	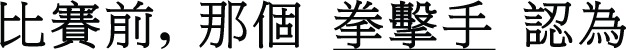 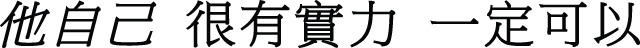 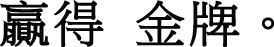 (20 sentences)	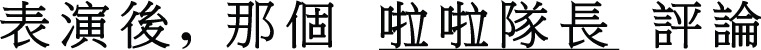 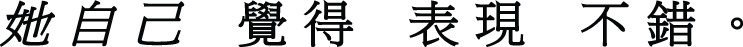 (20 sentences)
	(*Before the race, **that****boxer** considered **himself** very competent for winning the gold medal.*)	(*After the performance, **the****cheerleader** commented on **herself** for doing not badly.*)
**Gender incongruent**	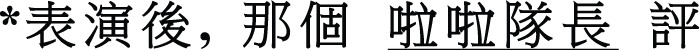 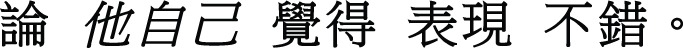 (20 sentences) (^*^After the performance, **the****cheerleader** commented on **himself** for doing not badly.)	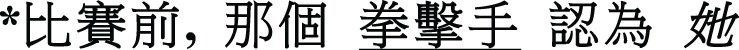 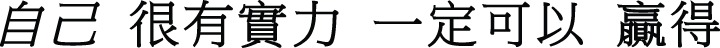 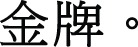 (20 sentences) (^*^Before the race, **that****boxer** considered **herself** very competent for winning the gold medal.)
**Fillers**	**Semantic match**	**Semantic mismatch**
	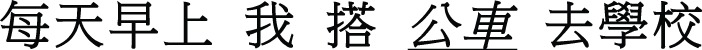 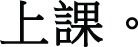 (40 sentences)	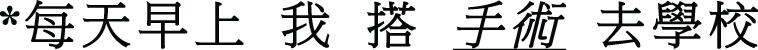 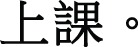 (40 sentences)
	(*Every morning, I take **the bus** to school.*)	(^*^Every morning, I take **the surgery** to school)

The sentence which is marked with an asterisk (

* *) is taken as antecedent-reflexive pronoun gender incongruent or semantic anomaly during data analysis*.

### Procedure

All the stimuli were presented in white letters on a black background. Each trial began with a fixation point “+” at the center of the screen for 500 ms, followed by a blank screen for 400 ms. Each word was presented for 400 ms followed by a blank screen for 400 ms. According to some previous studies (Ye et al., 2007; Jiang and Zhou, 2009), 400 ms (word) + 400 ms (blank) word presentation is natural and comfortable for Chinese readers. A variable inter-trial time interval (from 1700 to 3000 ms) appeared after each sentence.

Participants were comfortably seated in a sound-attenuated cubicle and were instructed to read each sentence silently and carefully. Their task was to answer yes/no comprehension questions by pressing one of the pre-designated buttons (“J” for “Yes” and “F” for “No”). A practice session with 12 trials was conducted before the main experiment. The main experiment was arranged in six blocks with five breaks. Each block contained 40 sentences. The 240 sentences were randomly presented, differently for each participant.

### Data recoding and analysis

Continuous EEG data (SynAmps2, NeuroScan) were acquired from 32 active electrodes mounted in a 66-channel Quick Cap. Electrodes were positioned according to the 10–20 system. The impedance was kept below 5Ω in each electrode. The sampling rate (A/D) was 500 Hz. The on-line reference electrode was set to be the left mastoid (M1) and we also recorded the right mastoid (M2). The signals were amplified with a bandpass of 0.05–100 Hz. The ground electrode was set between FPZ and FZ. HEOGs were placed at the outer canthi of the eyes and VEOGs were placed above and below the left eye in a bipolar montage.

The EEG raw data were re-referenced to the average activity of M1 and M2. The signal was bandpass filtered between 0.1 and 30 Hz. Epochs of interest were from −100 ms before the onset of the target word to 1000 ms after stimulus onset. Baseline correction was set from −100 ms to the onset of the target words. Trials with artifacts, such as eye blinks or saccades, or with activity exceeding ±120 μV were rejected. As a result, 5.6% of the trials were removed due to artifact rejection.

Except for trials with artifacts, all the correctly and incorrectly judged trials were included for statistical analysis. Data analysis focused on the mean voltage of each electrode within a time interval of interest after the onset of the target words in each participant. Based on the findings from previous ERP studies, four components, P200, LAN, N400, and P600 are used to index the processing correlates. The time intervals were chosen based on visual inspection of the averaged wave patterns.

Repeated measures ANOVAs were separately employed for electrodes in the midline region (anterior: average activity of Fz and FCz; central: Cz and CPz; and posterior: Pz and Oz) and in the lateral scalp (left anterior: average activity of Fp1, F3, F7, and FT7; left central: FC3, C3, CP3, and T7; left posterior: TP7, P3, P7, and O1; right anterior: Fp2, F4, F8, and FT8; right central: FC4, C5, CP4, and T8; and right posterior: TP8, P4, P8, and O2). A four-way repeated measure ANOVA was employed for the midline region considering *antecedent gender type* (definitional vs. stereotypical), *reflexive pronoun gender specificity* (default vs. specific), *gender congruity* (congruent vs. incongruent), plus the *latitude* topographical factor (anterior, central and posterior). For electrodes in the lateral scalp, a five-way repeated measures ANOVA was used: *latitude* and *lateral scalp* (left vs. right) were the topographical factors added to the three main factors. The Greenhouse-Geisser corrected *p*-value was used if the degree of freedom was larger than one. For interactions among the experimental factors and/or topographic factors, planned paired *t*-tests (with FDR adjusted *p*-value) were carried out mainly focused on the comparison of gender congruity respectively in each two levels of the main factors and/or separately in the topographic region to look for the location of the effect. Non-significant effects obtained from the planned paired *t*-tests following significant interactions are not reported in the data analysis.

## Results

### Comprehension questions

The average of participants' accuracy in the comprehension questions was 93%, ranging from 85 to 99%, showing that participants understood very well the sentences they read.

### ERPs on the reflexive pronouns

**Figure 1:**
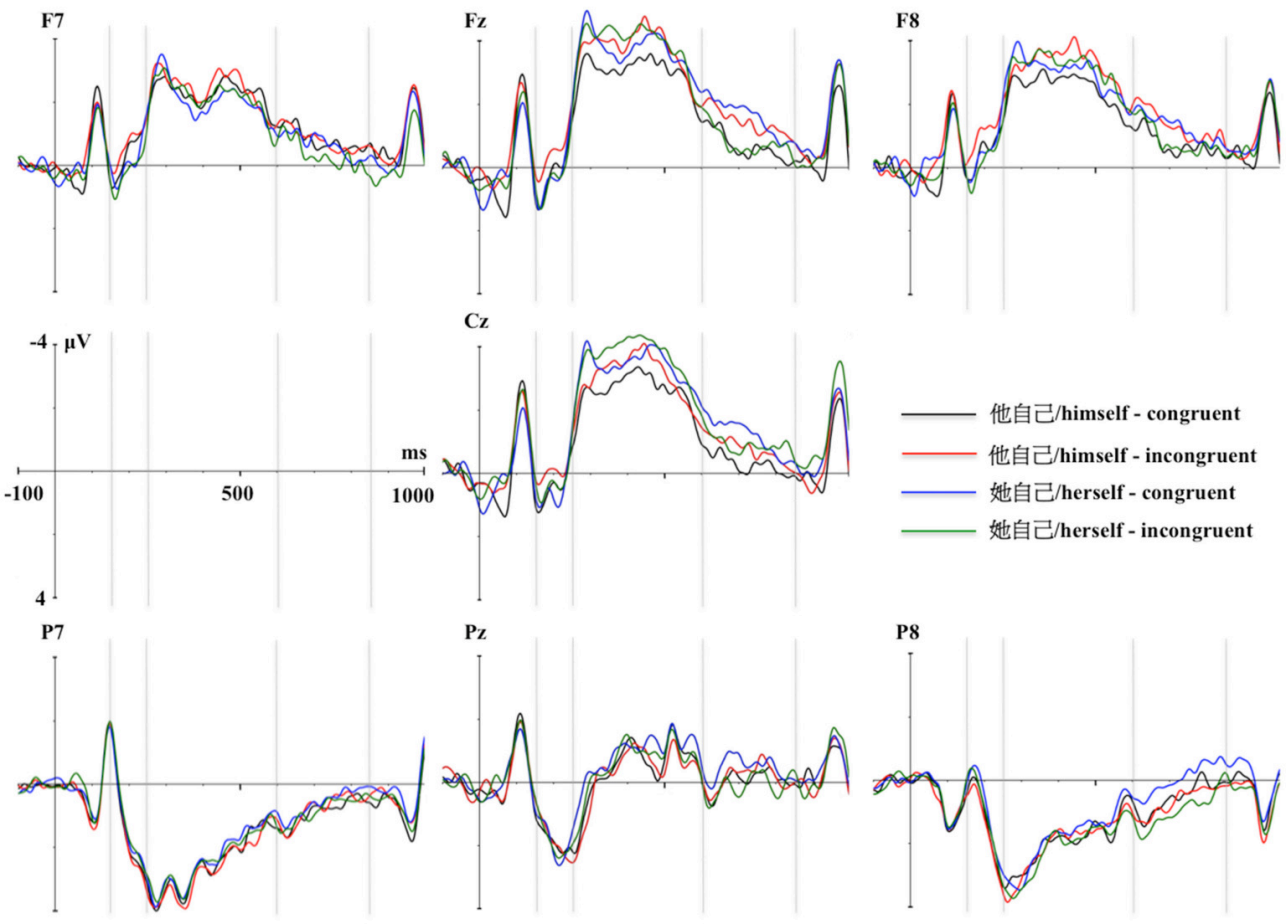
**Overall averaged brain activities of reflexive pronoun gender specificity (general vs. specific) by gender congruity (congruent vs. incongruent) in the representative electrodes**.

Figure 1 reports the grand average of the ERPs elicited by the two reflexive pronouns, taking into account gender congruity. Based on visual inspection of the overall ERP results (and supporting evidence in the literature), the time interval for the analysis of the P200 component was set as 150–250 ms after the onset of stimuli, that for the N400 component was 250-600, and that for the P600 component was 600–800. The repeated measures ANOVAs on the earlier time intervals did not show any statistically reliable effects (i.e., baseline correction: −100 to 0 ms; 0 to 150 ms).

### ERPs on the reflexive pronouns at the P200 time interval: 150–250 ms

The repeated measures ANOVA on the amplitude of the evoked activity for electrodes in the midline region showed an interaction among reflexive pronoun specificity, gender congruity, and latitude [*F*_(2, 78)_ = 4.43, *p* = 0.031]. The effect was found to be located in the mid-anterior region for 

/*himself* [congruent: 0.35 μV; incongruent: −0.33 μV; *t*_(39)_ = 2.02, *p* = 0.050] and no such effect was found for 

/*herself* (see Figure 1).

The repeated measures ANOVA for electrodes in the lateral scalp regions showed an interaction between *reflexive pronoun specificity* and *latitude* [*F*_(2, 78)_ = 9.34, *p* = 0.002] and an interaction among *reflexive pronoun specificity, gender congruity* and *latitude* [*F*_(2, 78)_ = 6.18, *p* = 0.010]. For the former interaction, the paired *t*-tests showed significant differences in the anterior region in which 

/*herself* was more positive than 

/*himself* [default: −0.49 μV; specific: −0.002 μV; *t*_(39)_ = −2.75, *p* = 0.009]. However, the second interaction did not reveal any relevant effects.

In this early time interval, a P200 *gender congruity* effect for the default reflexive pronoun (

**Figure 2:**
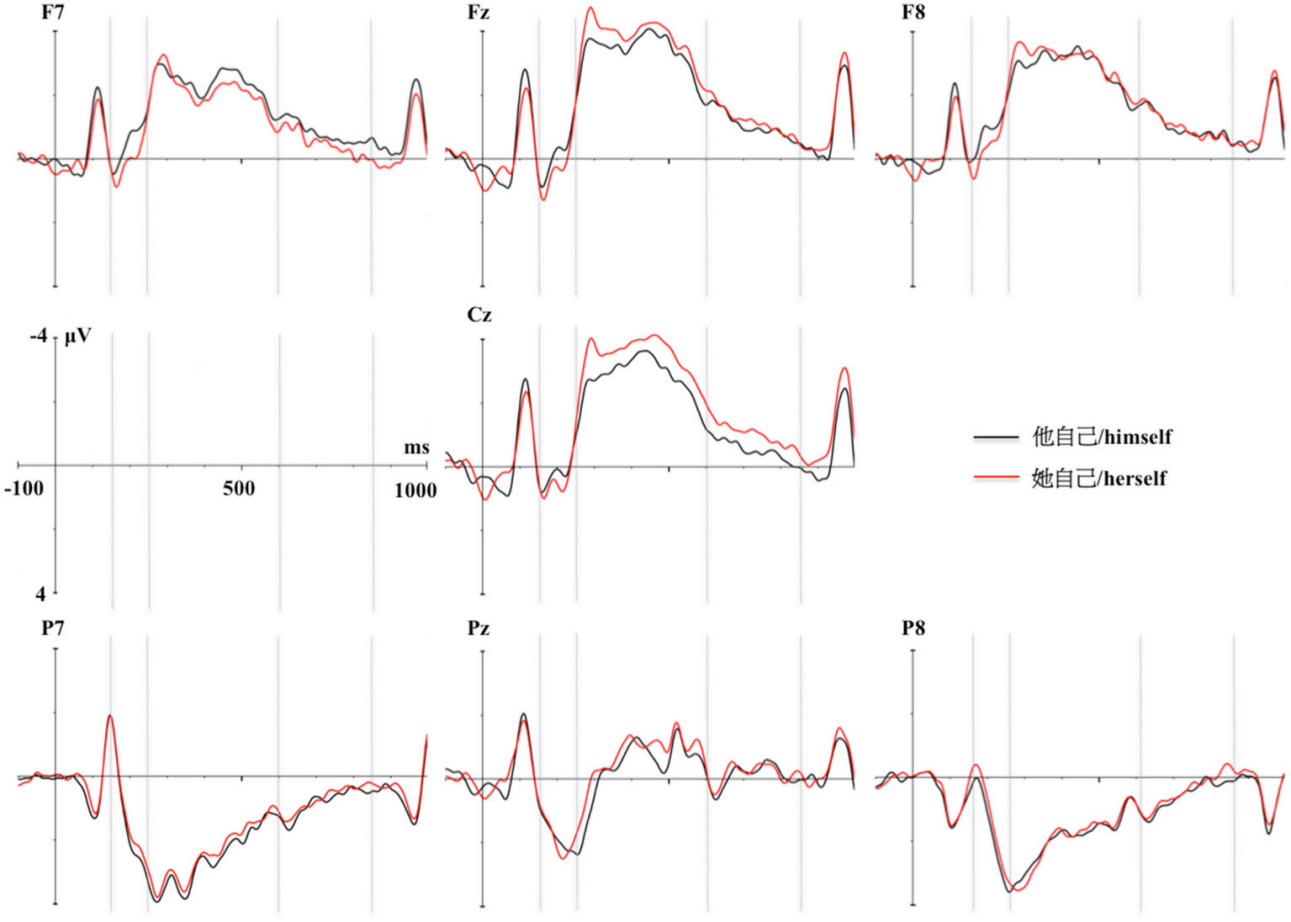
**The averaged brain activities separately presented by reflexive pronoun gender specificity (default: 

**Figure 3:**
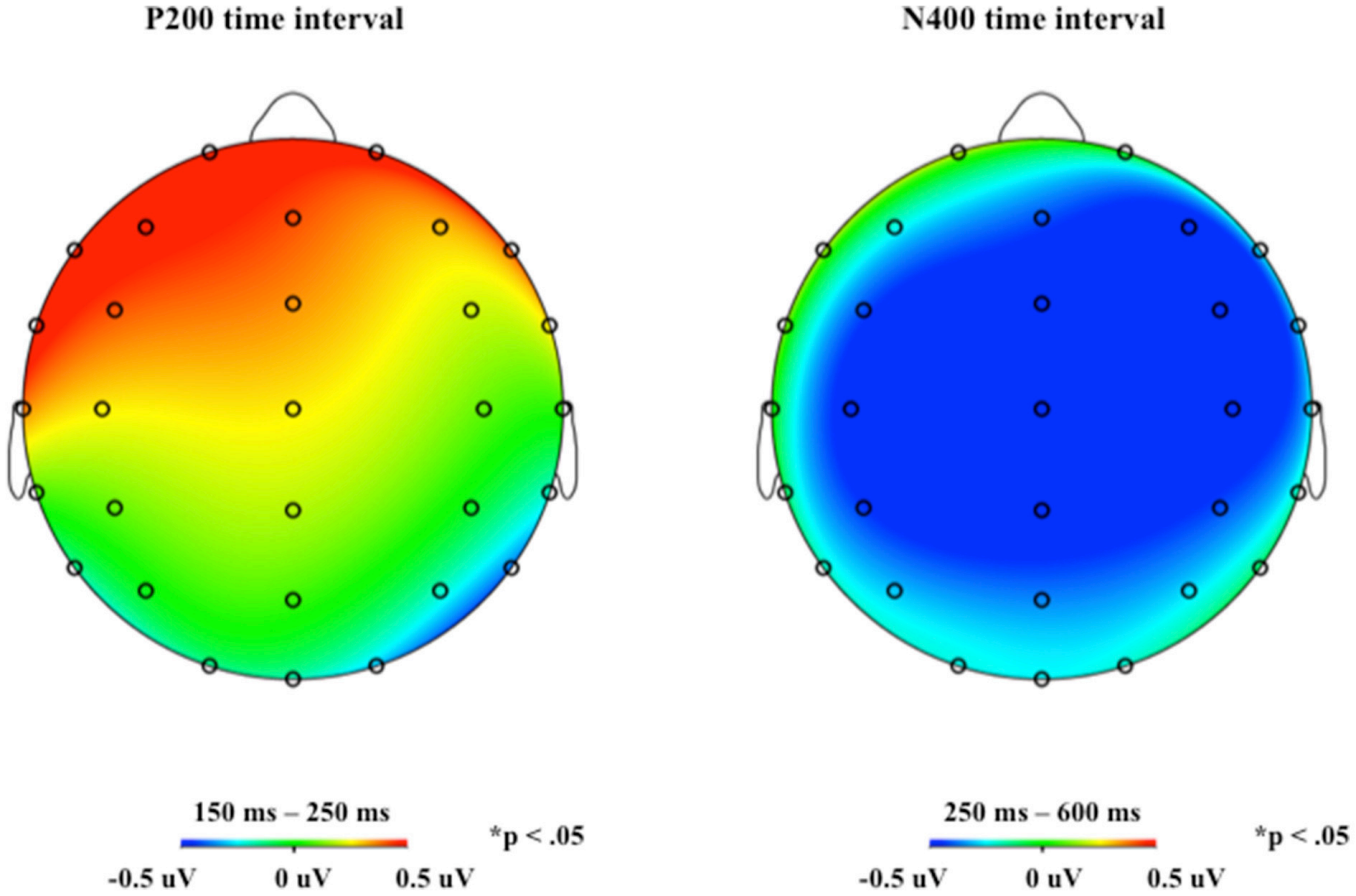
**Topographic distributions for the reflexive pronoun gender specificity effects (

**Figure 4:**
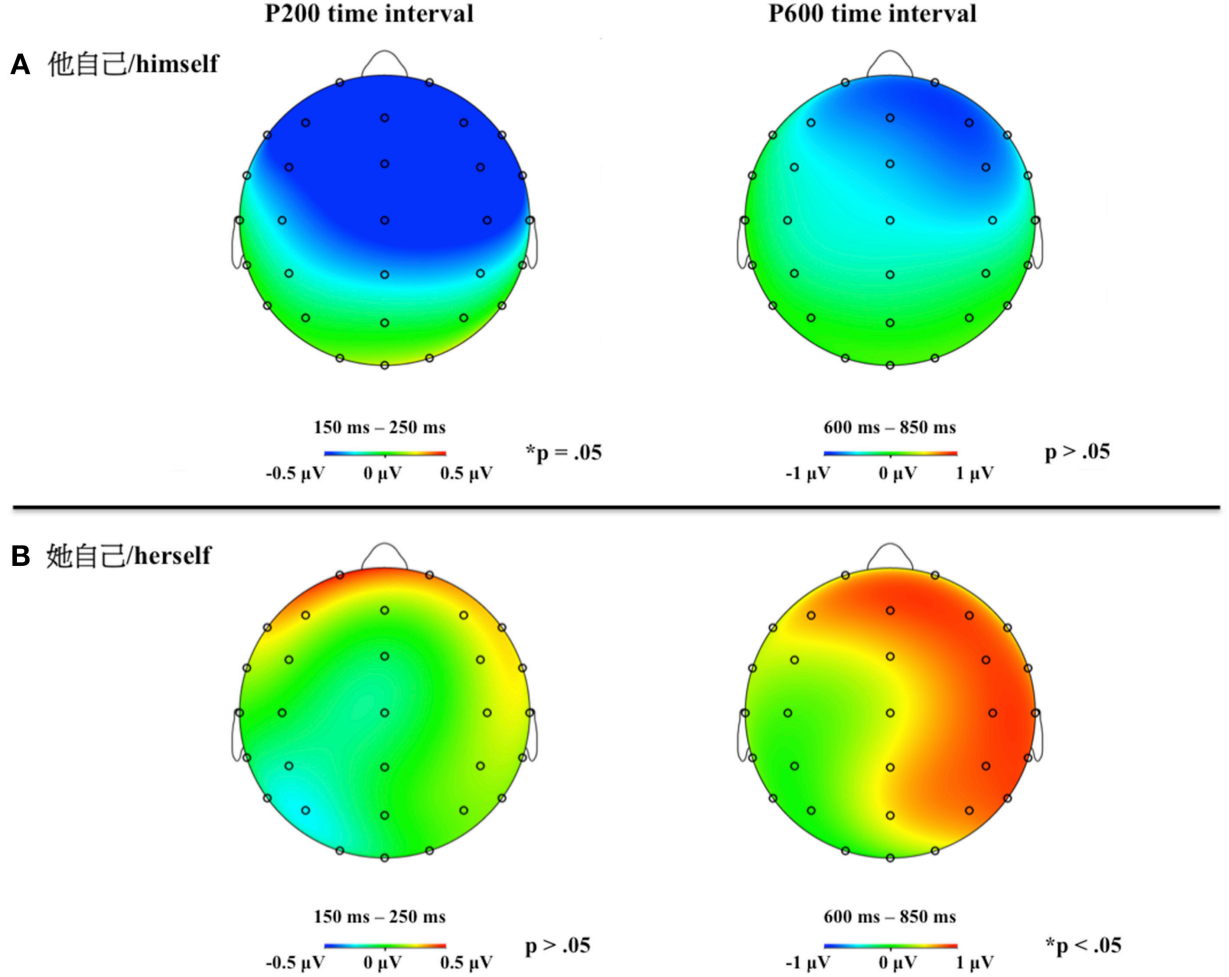
**Topographic distribution of the gender congruity effect (incongruent minus congruent) respectively for the two reflexive pronouns in the P200 and P600 time intervals**. Panel **(A)** represents effects for the general reflexive pronoun, 

/*himself*) in the mid-anterior region was observed (congruent > incongruent) (see Figures 1, 4) and a P200 *reflexive pronoun gender specificity* effect (specific > default) emerged in the lateral anterior region (see Figures 2, 3).

/*himself* vs. specific: 

/*herself*) in the representative electrodes**.

/*herself* minus 

/*himself*) respectively in the P200 and N400 time intervals**.

### ERPs on the reflexive pronouns at the N400 time interval: 250–600 ms

The repeated measures ANOVA on the amplitude of the evoked activity showed the following pattern: For electrodes in the midline region, a main effect of *reflexive pronoun specificity* revealed that 

/*herself* was significantly more negative than 

/*himself* [default: −1.56 μV; specific: −2.04 μV; *F*_(1, 39)_ = 7.33, *p* = 0.010] and an interaction between *gender congruity* and *latitude* [*F*_(2, 78)_ = 4.55, *p* = 0.028] were found. However, this interaction did not reveal any relevant effects in the paired comparisons.

For the electrodes in the lateral scalp, the ANOVA showed two interactions. The first one showed that the *antecedent gender type* interacted with *latitude*, [*F*_(2, 78)_ = 3.81, *p* = 0.035]. However, the paired comparisons did not show any significant differences. The second one was an interaction among *reflexive pronoun specificity, latitude*, and *lateral scalp* [*F*_(2, 78)_ = 4.19, *p* = 0.019]. The paired *t*-tests showed that 

/*herself* was significantly more negative than

/*himself* in the left central region [default: −1.37 μV; specific: −1.72 μV; *t*_(39)_ = 2.18, *p* = 0.035] and no relevant effects were found in other lateral regions.

Considering the whole experimental design, no interaction between the factors of interest emerged in the N400 time interval. Only an independent N400 *reflexive pronoun specificity* main effect was found in which the specific reflexive pronoun (

/*herself*) elicited more negative waveforms than the default (

/*himself*) (see Figures 2, 3).

### ERPs on the reflexive pronouns at the P600 time interval (600–800 ms)

The repeated measures ANOVA for electrodes in the midline region revealed three interactions. First, *antecedent gender type* was found to interact with *gender congruity* [*F*_(1, 39)_ = 4.53, *p* = 0.040], but the paired *t*-tests did not show any significant effects. Second, the *reflexive pronoun specificity* interacted with *gender congruity* [*F*_(1, 39)_ = 6.21, *p* = 0.017]. The planned paired *t*-tests showed that the incongruent 

/*herself* elicited more positive amplitude as compared to the congruent one [congruent: −1.02 μV; incongruent: −0.38 μV; *t*_(39)_ = −2.10, *p* = 0.042], but no such difference was found for 

/*himself* [congruent: -0.22 μV; incongruent: −0.61 μV; *t*_(39)_ = 1.26, *p* = 0.215]. Third, an interaction among *reflexive pronoun specificity, gender congruity*, and *latitude* [*F*_(2, 78)_ = 5.08, *p* = 0.015] emerged. The paired *t*-tests showed only a significant difference for incongruent 

/*herself* as compared to the congruent 

/*herself* in the anterior region [congruent: −1.77 μV; incongruent: −0.85 μV; *t*_(39)_ = −2.39, *p* = 0.022].

The repeated measures ANOVA for electrodes in the lateral scalp showed two interactions. The first one showed that the *reflexive pronoun specificity* interacted with *gender congruity* [*F*_(1, 39)_ = 8.84, *p* = 0.005]. The paired *t*-tests showed that the incongruent 

/*herself* elicited more positive amplitude as compared to the congruent one [congruent: −0.67 μV; incongruent: −0.12 μV; *t*_(39)_ = −2.56, *p* = 0.015], but no such effects emerged for 

/*himself* [congruent: −0.21 μV; incongruent: −0.46 μV; *t*_(39)_ = 1.31, *p* = 0.198]. The second interaction was among *reflexive pronoun specificity, gender congruity*, and *lateral scalp* [*F*_(1, 39)_ = 6.77, *p* = 0.013]. The paired *t*-tests revealed that the incongruent 

/*herself* was more positive than the congruent 

/*herself* in the right hemisphere [congruent: −0.86 μV; incongruent: -0.07 μV; *t*_(39)_ = −3.26, *p* = 0.002].

The most relevant finding in this P600 time interval was the interaction between *reflexive pronoun gender specificity* and *gender congruity*. While the amplitude for incongruent 

/*herself* was more positive than that for congruent 

/*herself*, no such effects emerged for

/*himself* (see Figures 1, 4).

/*himself*, and panel **(B)** represents effects for the specific reflexive pronoun, 

/*herself*.

## Discussion

The present study evaluates how the asymmetry of gender specificity encoded in characters for Chinese reflexive pronouns influences the processing of gender information during reflexive pronoun resolution in Chinese text. The results showed: First, two independent effects for 

/*herself*; a P200 effect (

/*herself* is more positive than 

/*himself*) and a N400 effect N400 effect (

/*herself* is more negative than 

/*himself*), supporting the assumption of 

/*himself* as the default pronoun. Second, a P200 gender congruity effect for 

/*himself* (congruent is more positive than incongruent) and a P600 gender congruity effect for 

/*herself* (incongruent is more positive than congruent) also emerged. The dissociation between 

/*himself* and 

/*herself* in the two time intervals provides relevant evidence of the asymmetry of gender specificity for anaphor resolution and suggests the distinct time courses involved in the processing of gender information during reflexive pronoun resolution. Most importantly, such dissociation further clarifies that the default pronoun 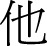/*he* is perceived as equally applicable to both genders instead of being a male-biased pronoun because no gender congruity effects emerged in the semantic-related N400 or the semantic/integration-related P600 time intervals.

### The processing of Chinese third person reflexive pronouns

As predicted, the less familiar and less frequent reflexive pronoun, 

/*herself* elicited more positive amplitude in the P200 time interval and more negative amplitude in the N400 time interval. The results thus reveal that the semantic radicals are processed at an early perceptual phase during Chinese orthographic recognition and lexical access is faster and easier for the default pronoun 

/*himself*, as compared to the specific 

/*herself*.

A straightforward explanation for the P200 and N400 specificity results could be that these arise because of frequency effects, due to the large difference in the word frequencies between the two characters (see Table 1 and Figure 2) (Dambacher et al., 2006; Kutas and Federmeier, 2011). According to Dambacher et al. (2006), the P200 could index processing differences resulting from word frequency comparisons (low frequency words elicit more positive amplitude) and the “*[…] lexical access was presumably completed for high-frequency words while low-frequency words were still being processed*” (p. 96). Meanwhile, N400 word frequency effects were reported for low frequency words as compared to high ones (Van Petten and Kutas, 1990; Dambacher et al., 2006), even in the context of word repetition in sentences (Van Petten et al., 1991). Although word frequency could explain the P200 and N400 effects reported here, the mechanism of how the two reflexive pronouns are processed and the dissociation of the two reflexive pronouns in the P200 and P600 time intervals are not clarified by this explanation, taking into account the co-reference between a reflexive pronoun and its antecedent within the structure of anaphor resolution.

Instead, the attention-related feature analysis (P200) and semantic expectation/predictability (N400) viewpoints may well explain the processing mechanism and the dissociation of the two reflexive pronouns during anaphor resolution. The P200 gender specificity effect could be interpreted as an attention-related mapping cost of the high similarity of graphic form in the two reflexive pronouns during Chinese character recognition (

/*himself* vs. 

/*herself*) (Luck and Hillyard, 1994; Liu et al., 2003). Liu et al. (2003) manipulated graphic similarity between a prime, 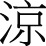/liáng/*cool*, and target, 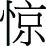/jīng/*startled*, in a pronunciation task and reported a P200 effect for the change in semantic radical. Following the attention-related feature analysis interpretation of Luck and Hillyard (1994), the authors concluded that the P200 effect is related to orthographic and phonological processing. This may possibly be the case in the 

/*himself*-

/*herself* contrast at the word level. As a default, the graphic form of 

/*himself* may be more familiar to participants and so make it easier to recognize/retrieve. In contrast, when 

/*herself* is encountered, participants need more effort to process the relatively less familiar graphic as compared to the default. This effect could be interpreted as basically driven by the semantic radicals encoded in the two reflexive pronouns.

Hung and Schumacher (2012, 2014) reported similar P200 effects in studies comparing the topicality (the amplitude for novel-topic was more positive than topic-shift, that was more positive than topic-continuity) and topic-worthiness (new topic was more positive than given topic) effects in Chinese discourse processing. The authors interpreted the P200 effect as reflecting early perceptual processing costs during discourse inference, taking into account the topicality or topic-worthiness. According to Hung and Schumacher (2014), the P200 “*[…] is likely to be a neural response to the involvement of selective attention that facilitates perceptual processing of an item that fulfilled contextually-induced expectation*” (p. 43). Due to the fact that similar sentence structures were employed in the present experiment, the default reflexive pronoun may be considered as a given topicality expected after every antecedent mentioned in the previous sentence fragment. The 

/*himself* as a default may account for this context-induced expectation of discourse inference. It could facilitate the perceptual processing and result in a neural brain response to its antecedent because it already fulfills the expectation driven by the contextual information. The more positive amplitude observed for 

/*herself* would accordingly reflect the processing of an unexpected item detected by the brain during early perceptual processing and result in a P200 effect, thus in line with Hung and Schumacher (2012, 2014).

On the other hand, the fact that the N400 effect (

/*herself* is more negative than 

/*himself*) could reflect the differential semantic expectation/predictability between the default (referring to any human) and the specific (referring to females only) reflexive pronouns. Since 

/*himself* is applicable to every mentioned antecedent, less negative amplitude is expected, either due to its all-inclusive semantic meaning (Hagoort et al., 2004; Lau et al., 2008; Rabovsky and McRae, 2014) or its more accessible orthographic form (see Delong et al., 2005: the expected article “a” or “an” in English, cf. Kutas and Federmeier, 2011).

### Anaphor resolution in a language without inflectional morphological gender markings

Taking into account the gender incongruity effects reported here, in terms of reflexive pronoun resolution, the present findings are consistent with Osterhout et al. (1997) in two respects. First, antecedent gender type (definitional as opposed to stereotypical gender) has no differential effect on the processing of gender information in anaphoric reflexive pronouns. Second, the processing cost for mismatches on gender emerges in the P600 time interval during reflexive pronoun resolution. According to Osterhout et al. (1997), although there is variability between definitional and stereotypical gender, as long as the gender information is activated (either male or female), the co-reference of this gender information during anaphor resolution should not vary by antecedent gender type. Thus, due to the syntactic constraints for definitional role nouns, and similar mismatch P600 effects found for both definitional and stereotypical role nouns, the authors concluded: gender information is “*[…] encoded within grammar*” (p. 282) and results in syntactic processing. Following Osterhout and colleagues, Kreiner et al. (2008, Experiment 1) further argued that the gender representation for such inference differed by noun types (i.e., definitional: lexical and stereotypical: pragmatic). Even so, Kreiner et al. (2008) reported no differences on the reflexive pronouns between sentences with definitional roles and those with stereotypical role nouns. Accordingly, our finding that no significant effects related to antecedent gender type emerged in any time interval very likely corresponds to what these authors described about the assignment of gender information regardless of antecedent noun type (definition or stereotypical) or how gender is represented by the antecedent (lexical or pragmatic). As long as the antecedent's gender is assigned, the activated gender is taken for the following anaphor resolution. However, in the present study, the P600 gender congruity effect found for 

/*herself* cannot be interpreted as reflecting syntactic processing, but is, rather, semantically-driven. As no morpho-syntactic gender agreement is required for Chinese anaphor resolution, the sentence remains grammatical when the character 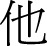 is replaced by 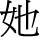. Only the meaning-based semantic radicals can account for the co-reference difficulty between antecedent and anaphor. This result is in line with Xu et al.'s (2013) proposal that the P600 effect in Chinese anaphor resolution reflects an integration difficulty based on semantic anomaly instead of reflecting processing difficulties with syntactic violations. This semantics-based interpretation of the P600 effect has already been offered by studies that did not involve any extra syntactic manipulations in their experiments (Münte et al., 1998; Kaan et al., 2000; Kaan and Swaab, 2003; Kolk et al., 2003; Kim and Osterhout, 2005; van Herten et al., 2005; Callahan, 2008; Van de Meerendonk et al., 2009; Molinaro et al., 2012). Thus, while findings in morphological gender languages (e.g., Osterhout et al., 1997; Kreiner et al., 2008, 2009) have shown the importance of syntactic gender agreement between antecedents and pronouns, the case of Chinese seems to be different. Reflexive pronoun resolution in written Chinese needs to rely on the semantic information unequally encoded in the orthographic forms of the characters, given that one orthographic form codes for a generic gender while the other codes specifically for a feminine representation. The generic gender encoded by the default pronoun 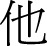/*he* is confirmed here to be a gender-neutral pronoun (appropriate to both genders) in line with the rating study that words with the radical 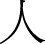/rén/*human* are rated as neutral (Cherng et al., 2009). Thus, mechanisms of co-reference of gender information between antecedents and reflexive pronouns in this study are modulated by the information carried by the semantic radicals denoting the different gender specificities of the pronouns.

The new finding here related to anaphor resolution is the P200 gender congruity effect found for the default reflexive pronoun 

/*himself*. This mid-anteriorly distributed P200 effect (congruent conditions elicited more positive amplitude as compared to incongruent ones) is different from the P200 reflexive pronoun gender specificity effect observed in the lateralized electrodes (

/*herself* is more positive than 

/*himself*, discussed in Section The Processing of Chinese Third Person Reflexive Pronouns), because the former effect is related to the gender of antecedents at sentence level. Thus, it seems that the conceptual processing of gender (the former P200) and the perceptual processing of character recognition (the latter P200) are processed in a similar time interval but are independent and associated with distinct scalp regions. Consistent with the attention-related mapping cost and context-induced expectation accounts, because of the similar sentence structures in the experimental stimuli, participants could expect the appearance of reflexive pronouns after encountering a proper name or a stereotypical role noun. If the antecedent is female, it is possible that participants are prepared for both reflexive pronouns, as both are applicable to a female antecedent. During the time interval in which the default (

/*himself*) is recognized, the evaluation of gender congruity is also easily accomplished because of its wide range of applicability. If the antecedent is male however, within this experimental context (because of the number of incongruent items of this nature presented), both the perceptual and conceptual processing systems might require more cognitive resources for encountering either 

/*himself* or 

/*herself*. The amplitude difference between the two contrasts (himself-congruent is more positive than himself-incongruent) might reflect such attention-related cost, related to prior contextual information (i.e., antecedent's gender here) deployed for processing the reflexive pronoun (Luck and Hillyard, 1994; Blanchet et al., 2007). In this case, the P200 is also sensitive to contextual information. On the other hand, when the specific reflexive (

/*herself*) is encountered, its recognition and retrieval are more complicated (as discussed in Section The Processing of Chinese Third Person Reflexive Pronouns) as compared to the default. It is possible that the word recognition is accomplished in the P200 time interval (i.e., the P200 effect for 

/*herself*) and the evaluation of gender congruity is delayed and resolved in a later time interval (i.e., the P600 effect).

Taking the main findings together, it is clear that 

/*himself* as a default with neutral gender is a critical feature in the perceptual and conceptual processing of gender information during Chinese reflexive pronoun resolution. Both types of processing rely on the semantic radicals encoded in the characters, suggesting the essential importance of the gender-based radicals to decoding of gender specificity. In terms of gender congruity effects, only when there is a clear mismatch (i.e., male/male-biased antecedent followed by the specific female reflexive), do our results support a two-stage model of anaphor resolution (Garrod and Sanford, 1994). As a whole, given the more familiar orthographic form (perceptual) and applicability to both genders (conceptual) of 

/*himself*, this pronoun may serve as a baseline during anaphor resolution. In addition, the ERP pattern for Chinese reflexive pronoun resolution confirms distinct time courses of processing for the two reflexive pronouns. While the default, 

/*himself*, is processed mainly at the early perceptual stage of character recognition and gender evaluation, the processing of the specific reflexive pronoun, 

/*herself*, lasts from the early perceptual stage (bonding stage, possibly including the N400 time interval) to the late integration stage (resolution stage). It is possible that there is more than one way to resolve anaphors. One is a two-stage model when the gender of an anaphor is specific and the mismatch is definite. The other is a one-stage processing model in which a default anaphor is eligible for every mentioned antecedent, as is the case of 

/*himself* reported in the present study. This does not mean that these results are specific to languages with default and specific distinctions on pronouns, as in Chinese. Instead, in addition to the well-established two-stage model for anaphor resolution (when pronouns have a specific gender), the one-stage model extends the description of anaphor resolution to when a pronoun can be used for both genders (in other words, a genderless pronoun). According to Siewierska (2013), genderless pronouns are used in 67% (254 out of 378) of world languages (such as Finnish, Turkish, Thai, Indonesian, Vietnamese or Maori …etc.). The results observed here may thus be relevant to the 67% languages with genderless pronouns.

### Conclusion

The non-symmetrical gender specificity of the Chinese characters for third person reflexive pronouns was studied during anaphor resolution. Independent P200 and N400 gender specificity effects confirmed processing differences resulting from the different gender specificity of reflexive pronouns (encoded in their semantic radicals) and also suggested the functional role of 

/*himself* as a default during anaphor resolution. During reflexive pronoun resolution, the two types of gender specificity interact with gender congruity respectively in the P200 (

/*himself*) and P600 (

/*herself*) time intervals. These results provide further evidence in support of the two-stage model of anaphor resolution only when there is an unambiguous mismatch between the antecedent and anaphor. The ERP patterns of the two reflexive pronouns also highlight the distinct time courses of anaphor resolution resulting from the two types of gender specificity. Overall, the findings in the present study demonstrate the importance of taking into account the asymmetry of gender specificity in Chinese third person reflexive pronouns, as well as confirming 

/*himself* as the default applicable to both genders.

## Author contributions

All authors listed, have made substantial, direct and intellectual contribution to the work, and approved it for publication.

### Conflict of interest statement

The authors declare that the research was conducted in the absence of any commercial or financial relationships that could be construed as a potential conflict of interest.

## Footnotes

^1^The term anaphor here refers to all kinds of anaphors in general that could refer back to an antecedent in previous text.

^2^According to Packard (2000) on Chinese word formation, the basic orthographic unit in Chinese written words is the character and generally one character represents one morpheme. From this point of view, Chinese has morphological gender markings, which are represented by individual characters/morphemes instead of inflections on a root. The term “morphological markings/cues,” however, usually refers to inflectional morphology.

^3^Typically, a written Chinese character represents one morpheme and one syllable and is composed of two parts: a semantic radical indicating the meaning and a phonological component that provides information about the pronunciation.

^4^Dictionary of Chinese Character Variants (2000). Available online at: http://dict.variants.moe.edu.tw/eng.htm (Accessed April 30, 2015).

^5^The plural is formed by adding another character, /men/, which is a collective marker, to either of these pronouns to form /tā men/*they* (male) or /tā men/*they*(female) (Li and Shi, 2000).

^6^The character /she was recorded as first appearing in  (Article of Jade, a manuscript written in the Yuan Dynasty), but the pronunciation and meaning is identical to /jiě/ meaning elder sister or ladies (Ling, 1989; Hua, 2012; Dictionary of Chinese character variants established by the Ministry of Education in Taiwan). The Yuan Dynasty was from 1271 A.D.-1368 A.D.

^7^Word List with Accumulated Word Frequency in Sinica Corpus (2005). Available online at: http://elearning.ling.sinica.edu.tw/eng_teaching_index.html (Accessed April 30, 2015).
